# READSCAN: a fast and scalable pathogen discovery program with accurate genome relative abundance estimation

**DOI:** 10.1093/bioinformatics/bts684

**Published:** 2012-11-28

**Authors:** Raeece Naeem, Mamoon Rashid, Arnab Pain

**Affiliations:** Pathogen Genomics Laboratory, Computational Bioscience Research Center, King Abdullah University of Science and Technology (KAUST), Thuwal-23955-6900, Kingdom of Saudi Arabia

## Abstract

**Summary:** READSCAN is a highly scalable parallel program to identify non-host sequences (of potential pathogen origin) and estimate their genome relative abundance in high-throughput sequence datasets. READSCAN accurately classified human and viral sequences on a 20.1 million reads simulated dataset in <27 min using a small Beowulf compute cluster with 16 nodes (Supplementary Material).

**Availability:** http://cbrc.kaust.edu.sa/readscan

**Contact:** arnab.pain@kaust.edu.sa or raeece.naeem@gmail.com

**Supplementary information:** Supplementary data are available at *Bioinformatics* online.

## 1 INTRODUCTION

The idea of computational subtraction of human host sequences to identify microbial sequences was first implemented on Amazon EC2 (Elastic Compute Cloud) environment in the form of software PathSeq (Kostic *et al.*, 2011). An alternative open source workflow available on desktop computers was recently provided by Rapid Identification of Non-human Sequences (RINS) (Bhaduri *et al.*, 2012). More recently, a platform called CaPSID (Borozan *et al.*, 2012) to store and visualize the identified non-human sequences was described. We present READSCAN a highly scalable and efficient tool to analyze ultra-high volume of data produced by the latest sequencers like Illumina HiSeq (http://www.illumina.com/systems/hiseq_systems.ilmn) that can produce 3–6 billion short reads in a single run.

READSCAN uses the data parallelism in the sequenced reads and effectively distributes the processing on multiple Central Processing Unit (CPU)s. READSCAN’s core alignment procedure on multiple known references is based on SMALT (H. Postingl 2012, personal communication; http://www.sanger.ac.uk/resources/software/smalt/) a fast and accurate short read mapper that works for a range of sequencing platforms (e.g. Illumina, Roche-454, Applied Biosystems-SOLiD). READSCAN is highly portable to work on a dual core laptop computer with as small as 2 GB memory to a large Beowulf cluster with 100 s of compute nodes. READSCAN reports the genome relative abundance (GRA) of those identified non-host microbial sequences implemented based on a proven finite mixture model and expectation maximization algorithm (Xia *et al.*, 2011). The results are ranked in the order of most to least abundant species grouped by National Center for Biotechnology Information (NCBI) taxonomical tree. The software performs an alignment-based assembly to report the length of the region covered by the reads and weighted mean length of such contigs produced as a result. This serves as a useful metric in assessing the true–positive results and also eliminates the need for an assembly program for microbial sequences with known reference genomes.

## 2 METHODS

The software first indexes the host and pathogen database sequences on a chosen *k*-mer value *r* based on the principle discussed in Baeza-Yates and Perleberg (BYP) (Baeza-Yates and Perleberg, 1996) 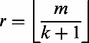



This *k*-mer value *r* allows us to detect the mutated sequences with maximum error or mutation rate of *k* in a string of length *m*.

**Fig. 1.**
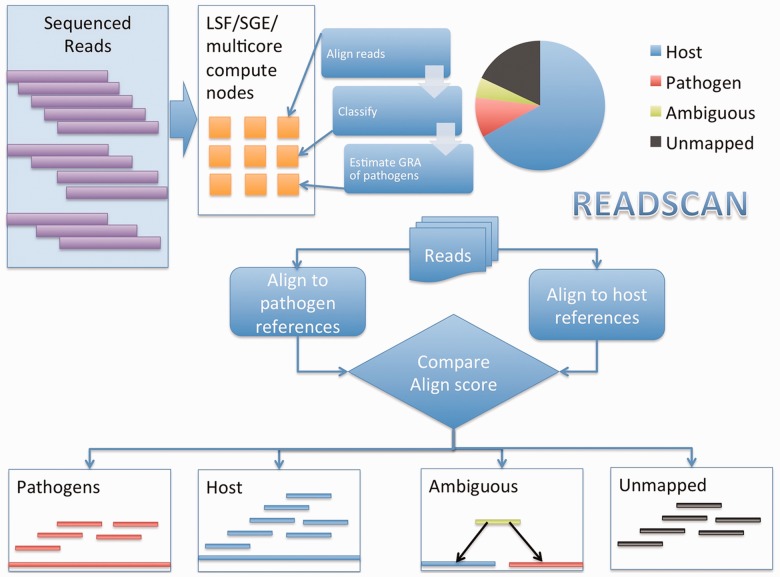
Schematic of READSCAN algorithm

The search phase as described in Figure 1 divides the input sequences into manageable chunks, and each chunk is processed in parallel. Each chunk is mapped against the host and pathogen references simultaneously using SMALT aligner. The result of the mapping procedure is filtered for per cent identity cut-off. The reads are then classified into several bins, namely, host, pathogen, ambiguous and unmapped. The classification is based on the alignment score reported by SMALT.

The directed acyclic graph representing the set of tasks and its dependencies is abstracted out, and the result is passed on to GNU make on a desktop computer and Makeflow (Yu *et al.*, 2010) on a multicore cluster to efficiently execute the tasks in parallel to speed up the overall throughput. The Makeflow abstractions are the key that make the program highly portable and execute directly without any modification on Load Sharing Facility (LSF), Sun Grid Engine and various other load levelers. The memory and resource requirements for the alignment tasks are computed using the formula provided by the SMALT aligner, and these values are passed to the appropriate job scheduler. SMALT-like other short read aligners have an inherent maximum limitation (Martin *et al.*, 2012) on the size of the database that can be indexed. This limitation is overcome by splitting up the database into manageable parts, such that each part does not exceed the random access memory limitation on a particular compute node. This helps the workflow to accommodate multiple human references to improve the accuracy of human reads removal and also multiple pathogen references grouped by taxon like bacteria, virus, protozoa and fungi. Choosing an appropriate chunk size can control the speed of the entire search phase.

Because of sequence similarity between reference sequences in the pathogen database the same read may map to multiple references in a non-unique mapping. Hence, the resulting statistics file is clustered by NCBI taxonomy tree and the GRA for particular species is reported as a sum of the GRA of all reference sequences of that species.

## 3 RESULTS

### 3.1 Performance of READSCAN on real dataset

We tested the performance of the READSCAN on a real dataset of RNA sequencing of 11 pair control and matched colorectal carcinoma samples (Castellarin *et al.*, 2012). READSCAN was able to detect the microbial flora present in the colorectal carcinoma and matched healthy tissues. The GRA values of different microbes in tumor and non-tumor samples have been shown as a heatmap (Supplementary Fig. S1), which clearly depicts the enrichment of *Fusobacterium nucleatum* sequences in nine tumor samples compared with their normal counterparts (one of the key findings presented in (Castellarin *et al.*, 2012).

Prostate cancer cell line SRR073726 (Prensner *et al.*, 2011) was analyzed, and READSCAN accurately reported the human papilloma virus (HPV) serotype 18 as the most abundant organism present in the sample with GRA of 68% and contig length of 953 bp in 39 min. RINS also matched the HPV serotype 18 with a contig of length 923 bp in 105 min. RINS matched 12 HPV reference sequences where READSCAN reported 18 HPV reference sequences grouped by HPV at the taxon level. The comparison was made on the same computer with exactly the same viral and human references.

### 3.2 Performance comparison—READSCAN, RINS and PathSeq on simulated dataset

**Fig. 2.**
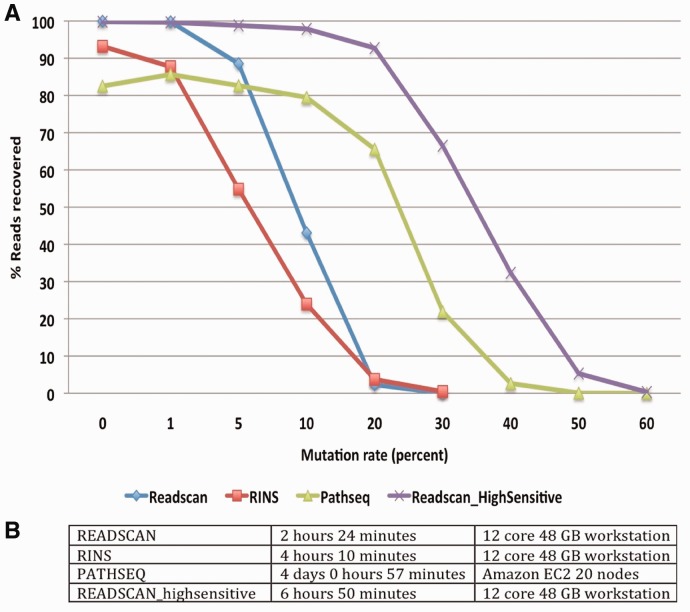
Performance comparison of READSCAN, RINS and PathSeq on simulated dataset

The simulated dataset was generated from human transcriptome and 12 viral genomes (Supplementary Methods). READSCAN outperformed RINS and PathSeq in recovering viral reads with different mutation rates (Fig. 2A). Also, READSCAN is much faster than RINS and PathSeq (Fig. 2B) in its default mode. By tuning alignment indexing and min-identity parameters, READSCAN’s high-sensitive mode achieved higher sensitivity with a trade off in specificity and time (Fig. 2). PathSeq achieved 100% specificity (ability to remove human reads) closely followed by READSCAN and RINS with 99.99% specificity in removing the human reads (Supplementary Fig. S2). To benchmark the scalability of READSCAN with added compute power, the same dataset was used on 1, 2, 4, 8 and 16 compute nodes where READSCAN scaled up linearly and completed the run in <27 min using 16 nodes (Supplementary Fig. S3).

## 4 CONCLUSIONS

READSCAN is a fast and accurate sequence search tool available on a variety of clusters and workstations designed to handle large next-generation sequencing datasets and detect non-target or pathogenic sequences.

*Funding*: KAUST faculty funding to A.P. supports this work.

*Conflict of Interest*: none declared.
